# Evaluating the impact of faculty performance appraisal systems on curriculum development and laboratory innovation in pharmaceutical education

**DOI:** 10.1186/s12909-026-09744-0

**Published:** 2026-06-26

**Authors:** Seeni Mohamed Jaleel Nilofer Fathima, Chidhambaram Muthu Velayutham

**Affiliations:** 1 Department of Science & Humanities, Sethu Institute of Technology, Viruthunagar, Tamil Nadu 626115 India; 2 Department of Management Studies, Anna University Regional Campus, Madurai, Tamil Nadu 625019 India

**Keywords:** Curriculum development, Pharmaceutical pedagogy, Faculty motivation, Laboratory innovation, FPAS, Curriculum design, Higher education India

## Abstract

**Background:**

Pharmaceutical education is undergoing significant transformation, requiring faculty to engage in curriculum innovation, technology-enhanced teaching, and advanced laboratory management. However, Faculty Performance Appraisal Systems (FPAS) in Indian pharmacy institutions, particularly in Tamil Nadu, continue to prioritize traditional quantitative metrics, such as publication counts, grant acquisition, teaching workload, and compliance-oriented performance indicators, often overlooking contributions to curriculum development and laboratory modernization. FPAS are commonly used institutional evaluation mechanisms in Indian higher education for faculty appraisal, promotion, and quality assurance. This misalignment may hinder pedagogical innovation and faculty motivation.

**Methods:**

A contextual and institutional analysis was conducted across pharmacy education settings in Tamil Nadu covering 12 institutions and 412 faculty respondents to examine existing FPAS frameworks and their alignment with contemporary curricular and laboratory expectations. A cross-sectional quantitative survey design was adopted. The study analyzed appraisal criteria, institutional practices, and faculty perceptions with particular focus on FPAS rigor, transparency, fairness, laboratory excellence, pedagogical innovation, and educational technology adoption, to identify gaps between evaluation mechanisms and educational innovation requirements. The analysis also aimed to generate evidence-based directions for strengthening appraisal practices.

**Results:**

The findings revealed that prevailing appraisal systems inadequately recognize curriculum redesign, pharmaceutical education technologies, and laboratory-based innovations. Overreliance on quantitative indicators and subjective assessments was associated with reduced faculty engagement, limited adoption of innovative teaching methodologies, and stagnation in curriculum advancement. Regression and structural equation modelling indicated significant positive associations between FPAS rigor and pedagogical innovation (β = 0.462, *p* < 0.001), FPAS transparency and laboratory excellence (β = 0.517, *p* < 0.001), and FPAS fairness and faculty motivation (β = 0.498, *p* < 0.001).

**Conclusion:**

The study proposes a restructured, multidimensional FPAS framework that formally integrates curriculum development outcomes, educational technologies, and laboratory excellence into faculty evaluation. By adopting transparent and developmental appraisal benchmarks, institutions can foster sustained pedagogical innovation and research-informed teaching. This approach offers practical guidance for academic leaders and policymakers aiming to strengthen curriculum quality in pharmaceutical education.

**Supplementary Information:**

The online version contains supplementary material available at 10.1186/s12909-026-09744-0.

## Introduction

The breakthrough in technological advancements and the changing social demands is rapidly transforming the fields of higher education, particularly in pharmaceutical sciences. In this evolving context, schools and other learning institutions should move beyond being inert depositories of information and function as dynamic institutions that foster professional competence, creativity, and pragmatic skills. The faculty members will be instrumental in this change since they are the ones who will define the learning experience and relevance of the curriculum. In the light of the changing industry needs and expectations on the students, systematic approaches to assessment of faculty performance is now critical towards the maintenance of accountability and policy improvement of quality of the institutions. Such performance assessment schemes must promote ongoing professional development and recognize different scholarly works. Most Indian universities continue to use rigid assessment mechanisms in which quantitative measures, such as the number of teaching hours and the number of publications are valued more than qualitative enhancement of the laboratory processes, curriculum and educational technology. Such an imbalance might lead to stagnation in laboratory and teaching standards and reduced faculty motivation, thereby affecting the quality of pharmaceutical education. In this context, ongoing reforms in pharmaceutical education, including accreditation requirements and quality assurance reviews by the pharmacy council of india (pci), national assessment and accreditation council (naac), and national board of accreditation (nba), require closer examination of faculty performance appraisal systems.

**Fig. 1:**
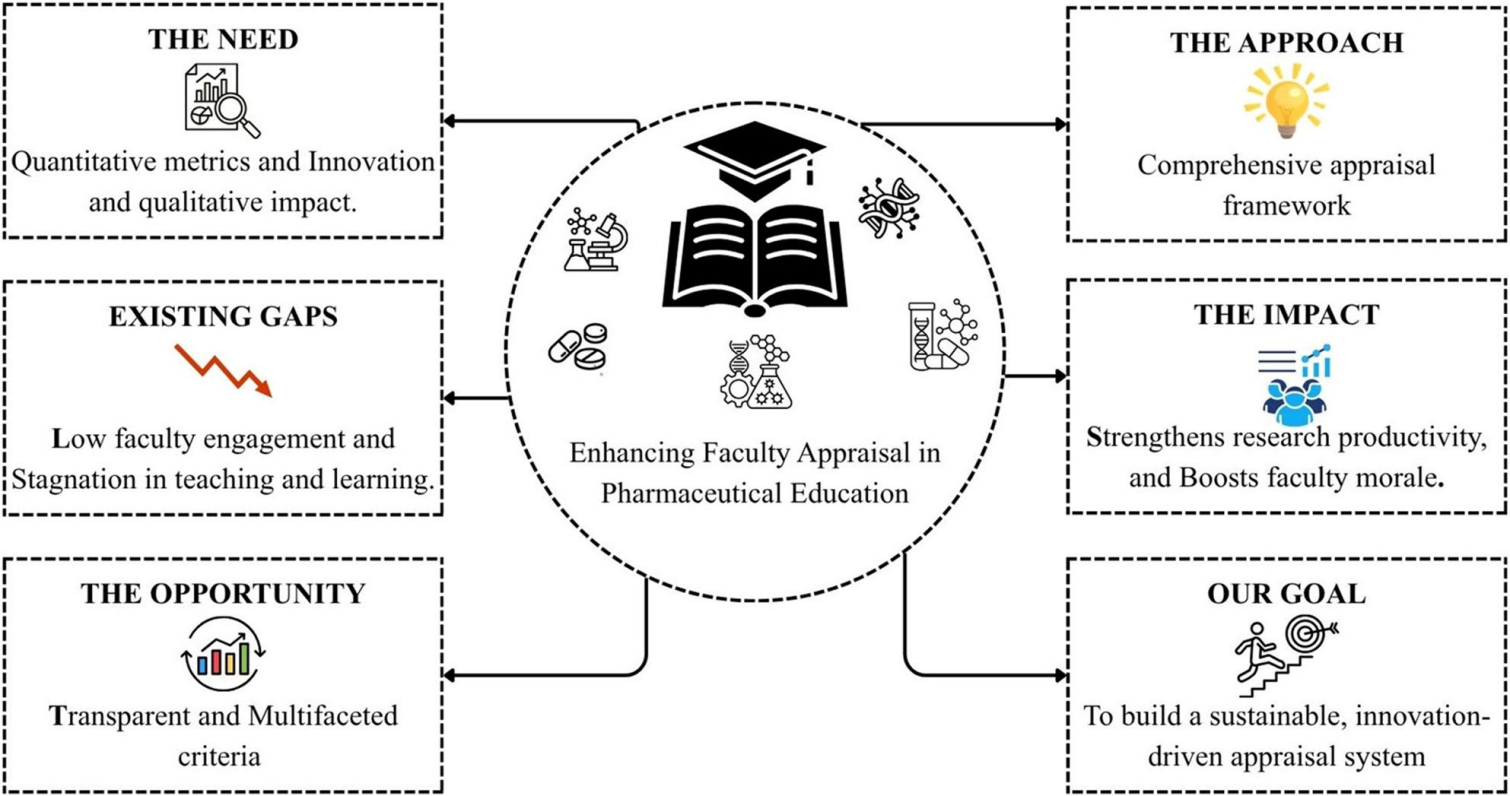
Faculty appraisal in pharmaceutical education

Modern pharmacy programs demand faculty members to teach in other fields, participate in new laboratory methods, and use digital and simulation-based learning media. Faculty appraisal systems may be effective in competitive settings such as Tamil Nadu, and may therefore, influence institutional objectives and teaching methods. Even though there is no empirical evidence of the impacts of these systems on laboratory performance and creativity training in the pharmaceutical sciences [1], has revealed their impact on academic culture and potential innovation. The Faculty Appraisal in pharmaceutical education was shown in (Fig. 1).

This paper acknowledges the necessity to establish appraisal systems as developmental initiatives and not administrative ones, and seeks to identify gaps in the existing evaluation systems with respect to the emerging requirements of pharmacy education. It aims at establishing the foundation of the creation of assessment theories that will actually encourage innovation and long-term academic success.

### Related work

Through the use of technology, competency-driven learning, and expanded curricula, pharmaceutical education has undergone tremendous change. However, higher education evaluation systems have remained largely unaltered. Instead of traditional lecturing, teaching excellence now emphasizes laboratory innovation, digital engagement, and adaptability. This reiterates the necessity of researching and assessing whether the performance of faculty is supportive or inhibitive of the innovative teaching in professional pharmacy education. To provide recommendations on just assessment systems that appreciate diverse input of contributions of the four mission areas of practice, teaching, research, and service especially through reviewing practice faculty evaluations [2].

Past investigations on pharmacy faculty have indicated that research output is frequently viewed to have had institutional focus as compared to teaching and lab-related services. Teaching assessment has generally been centered on the classroom provision compared to research assessment which has been inclined towards publications and grant procurement [1]. These trends have been linked to a lack of clarity in role expectations, job dissatisfaction, and decreased faculty engagement when evaluation criteria are perceived to be inconsistent with actual academic responsibilities [3]. Recent scholarship has hence stressed the necessity of appraisal systems that better capture the multidimensional nature of the roles of pharmacy faculty, such as teaching, laboratory practice, research and academic service [4].

To identify evidence, best practices, challenges, and recommendations for streamlining postgraduate pharmacy education, this systematic review examines the current state of the field. The scoping review was conducted in accordance with the PRISMA-ScR protocol. A total of 5,542 articles were identified, of which 36 met the inclusion criteria. The selected studies focused on curriculum and courses, training and skill development, and mentorship and assessment techniques. The review found that postgraduate pharmacy education would benefit from curriculum refinement, structured mentorship, strengthened skill-based training, and more effective assessment practices [5]. The main conclusions include the fact that curricula based on competencies and the focus on communication, critical thinking, and soft skills are essential. Academic demands and real-life needs are some of the challenges faced because of limited resources and the balance of academic demands and real-life demands [6, 7]. Nonetheless, it was commonly stated that despite these changes, evaluation procedures in higher education institutions remained unchanged [8]. Furthermore, laboratory innovation, digital engagement, and pedagogical adaptability, rather than just traditional lecturing, have become increasingly important for teaching excellence in pharmacy programs [9].

However, traditional metrics were still given precedence over curriculum innovation and laboratory restructuring in institutional mechanisms for assessing faculty performance. Long-term engagement was also decreased because professional development programs meant to upskill faculty were frequently carried out without related appraisal recognition [10]. Consequently, there was uneven support for faculty preparedness for long-term curriculum change amongst institutions. As a result, international reviews stressed how important it is to incorporate curriculum reform initiatives into official assessment frameworks [11]. Innovative pedagogy studies showed that student-centered instructional strategies greatly improved laboratory competency and conceptual understanding in pharmacy education [12].

Therefore, reviewing educator proficiency interventions, however, revealed that performance appraisal frameworks rarely included pedagogical training outcomes. Furthermore, faculty adoption of new teaching methodologies necessitated ongoing work and reflective practice—two things that were frequently dismissed as incidental [13]. Practices for assessment and instruction delivery have changed as a result of the increasing use of digital tools and artificial intelligence in pharmacy education [14].

Nevertheless, technology-enabled workshops and asynchronous learning programs were usually assessed as extracurricular rather than essential academic contributions. Additionally, research on digital health education revealed that uneven institutional incentives caused a wide range in faculty readiness. Thus, it was determined that appraisal systems that overlooked digital innovation were impeding the development of technology in pharmacy programs [15]. Performance evaluation systems had a significant impact on professional development paths in higher education, according to bibliometric analyses of educator evaluation practices. But according to systematic reviews, accountability was frequently prioritized over developmental feedback in appraisal processes [16].

Furthermore, regional studies from India showed that when evaluation results were viewed as arbitrary or unrelated to innovative teaching, faculty engagement decreased. Pharmacy education has placed a greater emphasis on interdisciplinary learning, digital readiness, and laboratory excellence according to global research syntheses [17]. However, it was uncommon for formal evaluation structures to institutionalize innovation-oriented indicators. Additionally, faculty contributions to technology-enabled learning environments continue to be underappreciated, according to reviews of innovations in digital health. Consequently, it was found that appraisal systems in pharmacy education were falling behind global educational priorities [18].

### Research gap and contribution of the present study

A lot of research has been done on curriculum innovation, digital transformation and pedagogical reform in pharmacy education but not much on the effect of faculty performance appraisal systems on these topics. The existing literature failed to take into account the relationships between innovative educational practices and measurement systems and treated them as independent variables. As a consequence of this oversight, a lack of knowledge exists regarding the effect of evaluation criteria on teaching innovation and laboratory excellence, specifically to pharmaceutical education in Tamilnadu.

This gap is filled by the current study, which adds to the body of literature with a framework specific to the current needs of pharmacy education by methodically evaluating the effects of performance appraisal systems on pedagogical innovation and laboratory excellence.

### Methodology

In this study, a cross-sectional research design with structured surveys is utilised to explore the effect of Faculty Performance Appraisal Systems (FPAS) on the excellence of laboratories and pedagogical innovation in pharmaceutical education. FPAS is the official institutional structure employed to assess faculty performance in terms of teaching performance, laboratory duty, research performance, compliance with legal requirements, and service to the academic community. It is applied in directing professional growth, making appraisal decisions, and ensuring quality assurance in institutions. The research design targeted a sample of laboratory, pedagogical, and institutional performance measures of faculty-level innovation results and appraisal mechanisms in a controlled higher education environment. The validity of the questionnaire was approved by experts as well as pilot testing before data collection.

### Data collection

A structured questionnaire was used to gather primary data from faculty members working in pharmaceutical sciences departments at private and deemed-to-be universities in Tamil Nadu. Convenience sampling was adopted to select respondents who were directly engaged in in classroom teaching including faculty members, curriculum developers, laboratory coordinators, and academic administrators. The selection of institutions was based on inclusion criteria such as institutional age, laboratory maturity, and and accreditation (NAAC/NBA).Exclusion criteria included newly established institutions, those with limited laboratory infrastructure, and institutions without recognized accreditation. A structured questionnaire was used to gather primary data from faculty members working in pharmaceutical sciences departments at private and deemed-to-be universities (institutions granted autonomous academic status by the University Grants Commission, India) in Tamil Nadu. A total of 12 institutions were included in the study. Convenience sampling was adopted to select respondents who were directly engaged in classroom teaching, including faculty members, curriculum developers, laboratory coordinators, and academic administrators. The selection of institutions was based on inclusion criteria such as institutional age, laboratory maturity (referring to the adequacy, functional stability, and established duration of laboratory infrastructure), and accreditation status (NAAC/NBA). The exclusion criteria were recently established institutions, those with limited laboratory infrastructure, and institutions that have not been recognized by accreditation. The period of data collection was January 2025 to March 2025. The questionnaire was formulated on the basis of previous literature on faculty appraisal research, quality assurance of pharmacy education and models of institutional evaluation. It was crafted to collect perceptions towards FPAS rigor, transparency in appraisal, innovation recognition, laboratory support, and pedagogical freedom. Among the 450 questionnaires sent out, 412 valid responses were received resulting in response rate of 91.6. Triangulation of responses of the faculty was done using secondary sources, including internal academic policy documents, and NAAC/NBA self-study reports and institutional appraisal guidelines. Table 1 shows the demographic profile of the respondents.

**Table 1:** Demographic profile of respondents

Variable Category	Sub-Category	Frequency	Percentage (%)
Gender	Male	238	57.8
	Female	174	42.2
Academic Designation	Assistant Professor	216	52.4
	Associate Professor	124	30.1
	Professor	72	17.5
Highest Qualification	M.Pharm	188	45.6
	Ph.D. (Pharmaceutical Sciences)	224	54.4
Core Teaching Domain	Pharmaceutics	108	26.2
	Pharmaceutical Chemistry	94	22.8
	Pharmacology	86	20.9
	Pharmacy Practice	78	18.9
	Pharmaceutical Analysis	46	11.2
Laboratory Teaching Load	≤ 6 h/week	96	23.3
	7–12 h/week	214	51.9
	> 12 h/week	102	24.8
Accreditation Exposure	Pharmacy Council of India (PCI) only	168	40.8
	Pharmacy Council of India (PCI) + National Assessment and Accreditation Council (NAAC)	156	37.9
	pharmacy council of india (PCI) + national assessment and accreditation council (NAAC) + national board of accreditation (NBA)	88	21.3

Note: In pharmacy institutions, laboratory teaching commonly ranges between 7 and 12 hours per week; therefore, a laboratory teaching load of >12 hours per week may be regarded as relatively high, but it is not uncommon in laboratory-intensive programmes

Note: In pharmacy institutions, laboratory teaching commonly ranges between 7 and 12 h per week; therefore, a laboratory teaching load of > 12 h per week may be regarded as relatively high, but it is not uncommon in laboratory-intensive programmes.

### Data analysis

The descriptive statistics (mean and standard deviation) were then used to summarise the faculty perceptions to the Faculty Performance Appraisal System (FPAS) structure and innovation-related incentives. Cronbach alpha was used to test the reliability of the questionnaire to establish the internal consistency of the items contained in each construct. The alpha of the Cronbach test was used to test the hypothesis that two or more items in the questionnaire were used to measure the same construct they should be measuring. The coefficient value of 0.70 and more was regarded to be good in terms of reliability. After assessing reliability, inferential statistical methods, such as correlation, regression, and mediation analysis, were used to test the relationships that existed between appraisal rigor, laboratory excellence, pedagogical innovation, and faculty motivation. To establish whether the observed relationships were significant or not, a significance level of *p* < 0.05 was used in all the statistical tests.

### Conceptual framework

**Fig. 2:**
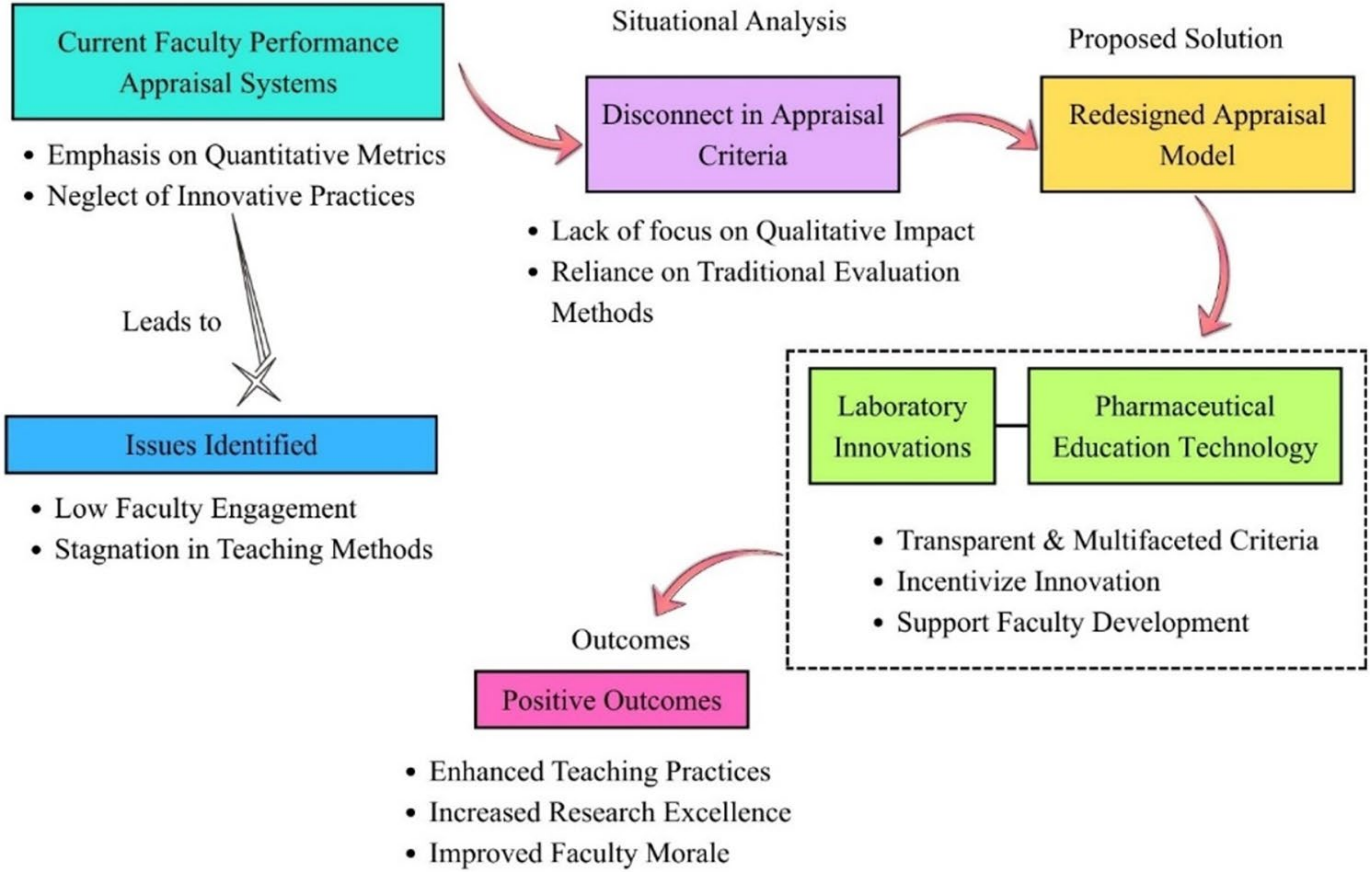
Conceptual framework

Faculty roles in this study are not restricted to teaching, but also to design of labs, compliance with regulations, and research–oriented academic activities. The framework identifies FPAS, rigor, transparency and sensitivity to innovation as key factors influencing pedagogical innovation and laboratory excellence. Examples of pedagogical innovation include curriculum redesign, use of digital tools, problem-based learning, and outcome evaluations. Modern equipment, adherence to safety regulations, creative experiment design, and conformity to industry and regulatory standards are all signs of a top-notch laboratory. Innovation can be facilitated or hindered by FPAS; excessively quantitative evaluation standards may ignore qualitative contributions linked to educational innovation, and laboratory advancements favoring conformity over originality. Figure 2 analyses the conceptual framework of this research.

Systems that acknowledge technological developments in education and laboratory innovation, on the other hand, promote ongoing improvement. Extrinsic factors by the accrediting bodies, like NAAC and NBA, are perceived to act as contextual moderators that subject FPAS to strict frameworks. The framework provides the basis of future studies and policy formulation in pharmaceutical higher education by defining a pathway that connects the issues of faculty drive and rigor of appraisal, and promotes academic innovation and laboratory superiority.

### Hypothesis development

**Fig. 3:**
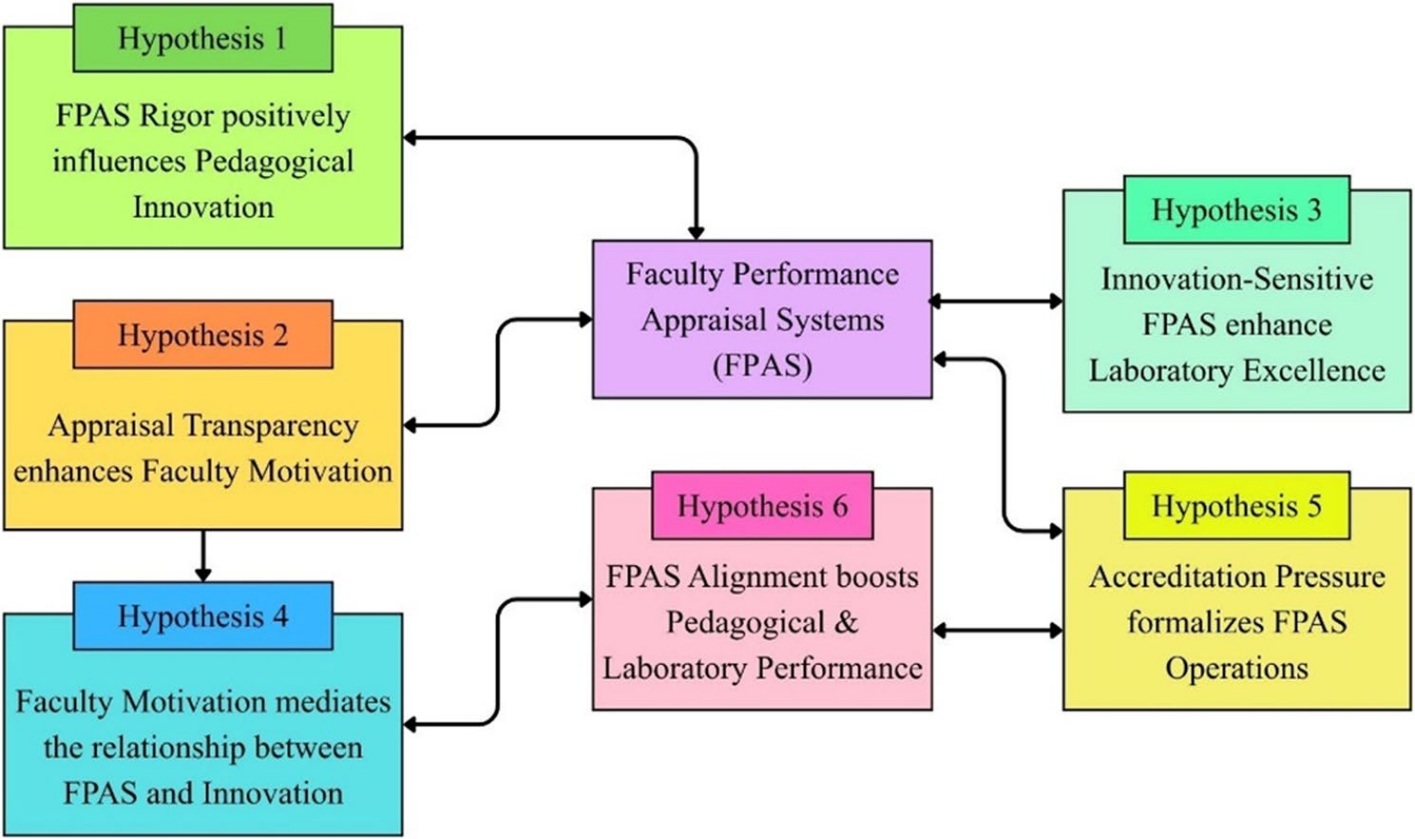
Hypothesis analysis

The hypothesis of this research is given in (Fig. 3):

### Faculty performance appraisal rigor and pedagogical innovation

#### Hypothesis 1: faculty performance appraisal rigor has a significant positive influence on pedagogical innovation in pharmaceutical education.

Pedagogical innovation needs to be followed by adherence to the outcome-based education frameworks, careful planning, and curriculum mapping to apply to the regulated fields, like the pharmaceutical sciences. The FPAS rigor holds faculty accountable and directs them, encouraging a clear framework through the use of defined performance indicators, regular assessments, and expectations. When systems of evaluation focus on more than administrative metrics, faculty are more inclined to employ tech-enabled pedagogy, problem-based learning, simulation-driven instruction, and curriculum redesign.

### Appraisal transparency and faculty motivation

#### Hypothesis 2: transparency in FPAS positively affects faculty motivation within pharmaceutical institutions

Transparency in evaluation systems eliminates the perceived subjectivity and information asymmetry, which eradicates the impediments to faculty participation in higher education. Unclear expectations of appraisals in pharmaceutical organizations can decrease motivation and promote a risk-averse attitude.

### Innovation sensitivity of FPAS and laboratory excellence

#### Hypothesis 3: FPAS, which specifically identifies laboratory innovation help to boost laboratory excellence

The pharmaceutical education laboratories demand infrastructure and innovative faculty involvement in the design and implementation of safety measures and experimentation. These are the qualitative factors that are mostly ignored in the conventional appraisal systems. Technical creativity can be identified with the help of FPAS models that incorporate the measures of innovation, like industry-oriented applied modules and modernization projects. By enhancing faculty to improve, this knowledge boosts student competency, improves the results of experiments, and ensures the attainment of pharmaceutical standards.

### Faculty motivation as a mediating mechanism

#### Hypothesis 4: The relationship between FPAS characteristics and pedagogical innovation is mediated by the effect of faculty motivation

The FPAS structures indirectly affect the outcome of innovation through the motivation of the faculty, yet have an effect on the expectations of the institutions. Systems of fair and development-based appraisals that are effective improve the motivation of the teachers and thus their commitment and self-efficacy. These incentives will motivate the faculty to embrace new pedagogical approaches and the application of technology that will aid in innovation in education. It brings out the point that motivation is not only the outcome of the evaluation process but also dynamic in terms of how the institutional controls and the innovation in education are related to one another.

### Accreditation pressure and FPAS formalization

#### Hypothesis 5: NAAC and NBA accreditation pressure have a positive effect on the formalization of FPAS in pharmaceutical institutions

According to the hypothesis, the systemic driver is the external regulatory pressure, which increases the rigor of FPAS and the uniformity of the procedures, mainly in the technical-oriented areas, where instructions and quality of the laboratory are questioned.

### FPAS alignment and integrated educational excellence

#### Hypothesis 6: alignment between FPAS criteria and pharmaceutical education objectives positively influences combined pedagogical and laboratory performance

Teaching excellence in pharmaceutical sciences results in the use of excellent laboratory techniques alongside successful teaching. The lack of alignment between faculty efforts and learning objectives causes a spread of their efforts when FPAS requirements bequeath compliance to important innovation. Conversely, assessment schemes that particularly align assessment measures with faculty expertise building, lab proficiencies, and teaching plan results harmonize faculty behavior and school mission. Due to this congruency, the quality of the pharmaceutical education is holistically enhanced because the teaching approaches enhance the laboratory excellence and vice versa.

### Statistical techniques and analytical procedures

The testing of the hypotheses and the empirical validation of the proposed conceptual model were done with the help of preliminary data validation and the inferential and causal tests based on the developed multivariate statistical analysis framework. To generalize respondent features and investigate the data distribution patterns, descriptive statistics began the analytical process. The scale stability was determined through assurance of measurement scales internal consistency and reliability before testing the hypothesis. After this, the direct relationships between FPAS traits, faculty motivation, pedagogical innovation, and laboratory excellence were examined with the help of correlation and regression analysis. Indirect effects were analyzed through mediation analysis and significance test, and explained variance was used to measure the sufficiency of the model.

### Reliability and internal consistency analysis

Reliability and internal consistency analysis were conducted to examine whether the questionnaire items measured each construct consistently. Cronbach’s alpha was used to assess the degree of internal consistency among the items within each construct. A coefficient value of 0.70 or above was considered acceptable, indicating satisfactory reliability for further statistical analysis. In the present study, the reliability values exceeded the accepted threshold, confirming that the measurement scale was suitable for subsequent correlation, regression, and structural equation modelling analyses. Equation 1 [19] expressed as

1 $$\:\alpha\:=\frac{k}{k-1}\left(1-\frac{\sum\limits_{i=1}^{k}{\sigma\:}_{i}^{2}}{{\sigma\:}_{T}^{2}}\right)\:$$

where $\:k$represents the number of items, $\:{\sigma\:}_{i}^{2}$denotes item variance, and $\:{\sigma\:}_{T}^{2}$indicates total scale variance.

### Correlation analysis

To find out how strongly and in which direction FPAS dimensions, faculty motivation, pedagogical innovation, and laboratory excellence are related Pearsons correlation analysis was performed. Before regression modeling, this analysis offered initial insights into linear relationships and multicollinearity risks. The Eq. 2 [20] was

2 $$\:r=\frac{\sum\:(X-\stackrel{\prime }{X})(Y-\stackrel{\prime }{Y})}{\sqrt{\sum\:(X-\stackrel{\prime }{X}{)}^{2}\sum\:(Y-\stackrel{\prime }{Y}{)}^{2}}}$$

Correlation coefficients within acceptable bounds supported the feasibility of regression-based causal analysis.

### Direct effect regression analysis

Multiple linear regression models were used in order to test the direct effects that were suggested in Hypotheses 1, 2, 3, and 6. While FPAS rigor, transparency, innovation, sensitivity, and alignment were independent predictors, pedagogical innovation and laboratory excellence were considered dependent variables. Eq. 3 [21] was expressed as

3 $$\:Y={\beta\:}_{0}+{\beta\:}_{1}{X}_{1}+{\beta\:}_{2}{X}_{2}+\cdots\:+{\beta\:}_{n}{X}_{n}+\epsilon\:$$

where $\:Y$denotes the outcome variable, $\:{X}_{n}$represents FPAS-related predictors, $\:{\beta\:}_{n}$indicates regression coefficients, and $\:\epsilon\:$captures the error term.

### Mediation analysis

A mediation framework that followed known causal steps was used to test the mediating function of faculty motivation as suggested in Hypothesis 5 . The analysis looked at whether faculty motivation was an indirect way that FPAS traits affected pedagogical innovation. Eq. 4 [22] illustrates as

4 $$\:M=aX+{\epsilon\:}_{1}Y={c}^{{\prime\:}}X+bM+{\epsilon\:}_{2}\:$$

where $\:X$represents FPAS characteristics, $\:M$denotes faculty motivation, $\:a$and $\:b$are path coefficients, and $\:{c}^{{\prime\:}}$represents the direct effect after accounting for mediation.

## Results and discussion

This section looks at how Faculty Performance Appraisal Systems (FPAS) influence pedagogical innovation, laboratory excellence, and faculty motivation within the pharmaceutical education institutions in Tamil Nadu. It uses hypothesis-based statistical tests to investigate both direct and mediated associations in an accreditation-oriented study. The discussion connects the empirical results with the regulatory and theoretical views on appraisal-based academic practices.

### Descriptive statistics of FPAS and pharmaceutical pedagogical constructs

**Fig. 4:**
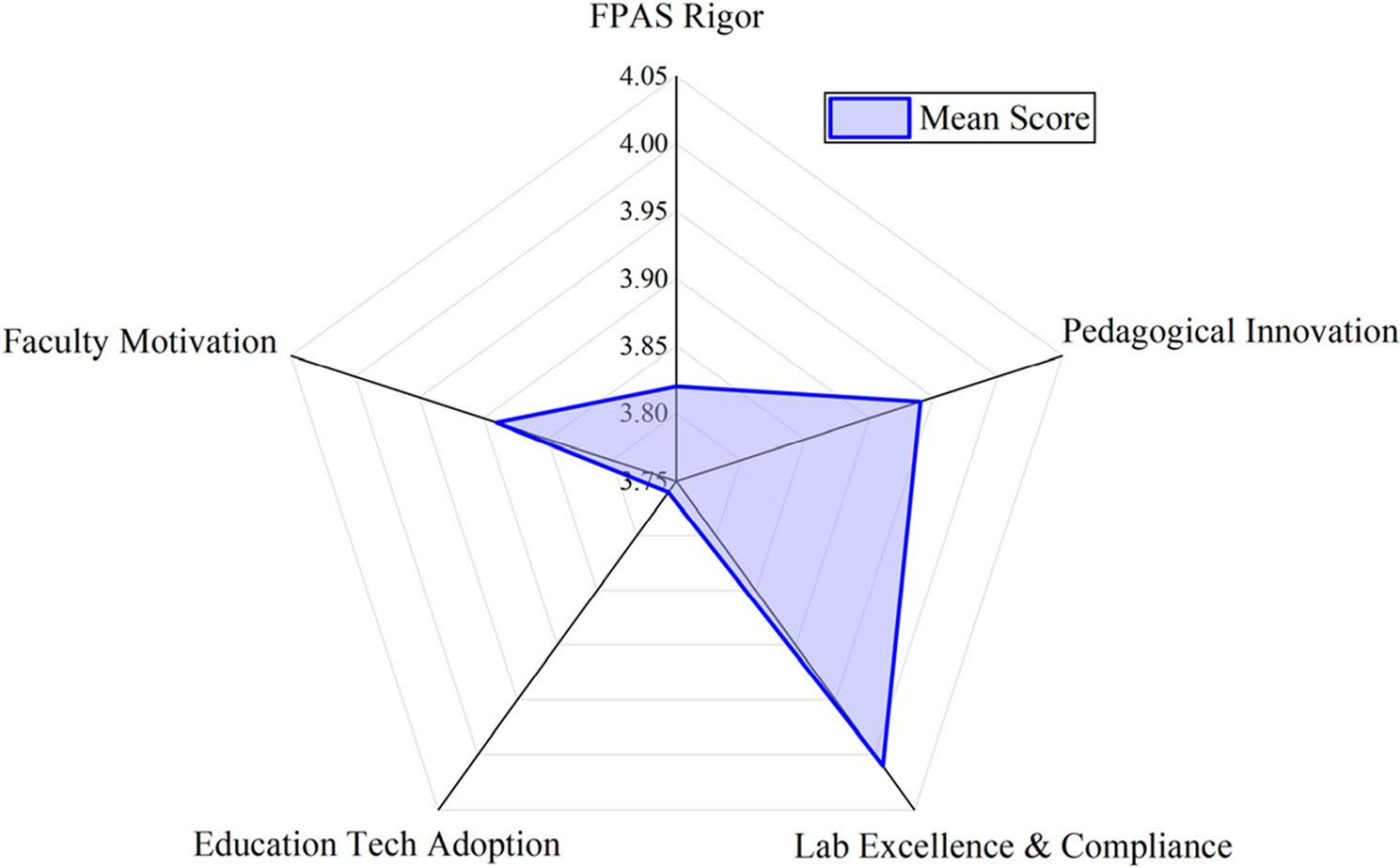
Baseline descriptive measures of FPAS and pedagogy

Table 2; Fig. 4 presented the descriptive statistics of the major pharmaceutical FPAS and innovation constructs. The mean scores indicated generally positive perceptions across all dimensions. Laboratory Excellence and Compliance recorded the highest mean score (M = 4.01, SD = 0.64), followed by Pedagogical Innovation in Pharmacy (M = 3.94, SD = 0.68) and Faculty Motivation in Lab-Driven Roles (M = 3.89, SD = 0.69). FPAS Rigor showed a favourable mean score (M = 3.82, SD = 0.71), while Pharmaceutical Education Technology Adoption had the comparatively lowest, though still positive, mean score (M = 3.76, SD = 0.73), indicating increasing but not uniform levels of technology integration.

**Table 2:** Descriptive statistics of pharmaceutical FPAS and innovation constructs

Construct	No. of Items	Mean	Std. Deviation	Skewness	Kurtosis
FPAS Rigor (Pharma-Specific Criteria)	6	3.82	0.71	−0.46	0.38
Pedagogical Innovation in Pharmacy	5	3.94	0.68	−0.52	0.41
Laboratory Excellence & Compliance	6	4.01	0.64	−0.61	0.55
Pharmaceutical Education Technology Adoption	5	3.76	0.73	−0.33	0.29
Faculty Motivation (Lab-Driven Roles)	4	3.89	0.69	−0.48	0.36

The skewness values ranged from − 0.33 to − 0.61, indicating a slight negative skew and suggesting that respondents tended to report higher levels of agreement. The kurtosis values ranged from 0.29 to 0.55, remaining within acceptable limits and indicating the absence of extreme departures from normality. These results suggested that the data were approximately normally distributed and were therefore suitable for subsequent parametric analyses.

### Hypothesis 1testing: FPAS rigor and pedagogical innovation in pharmaceutical education

**Fig. 5:**
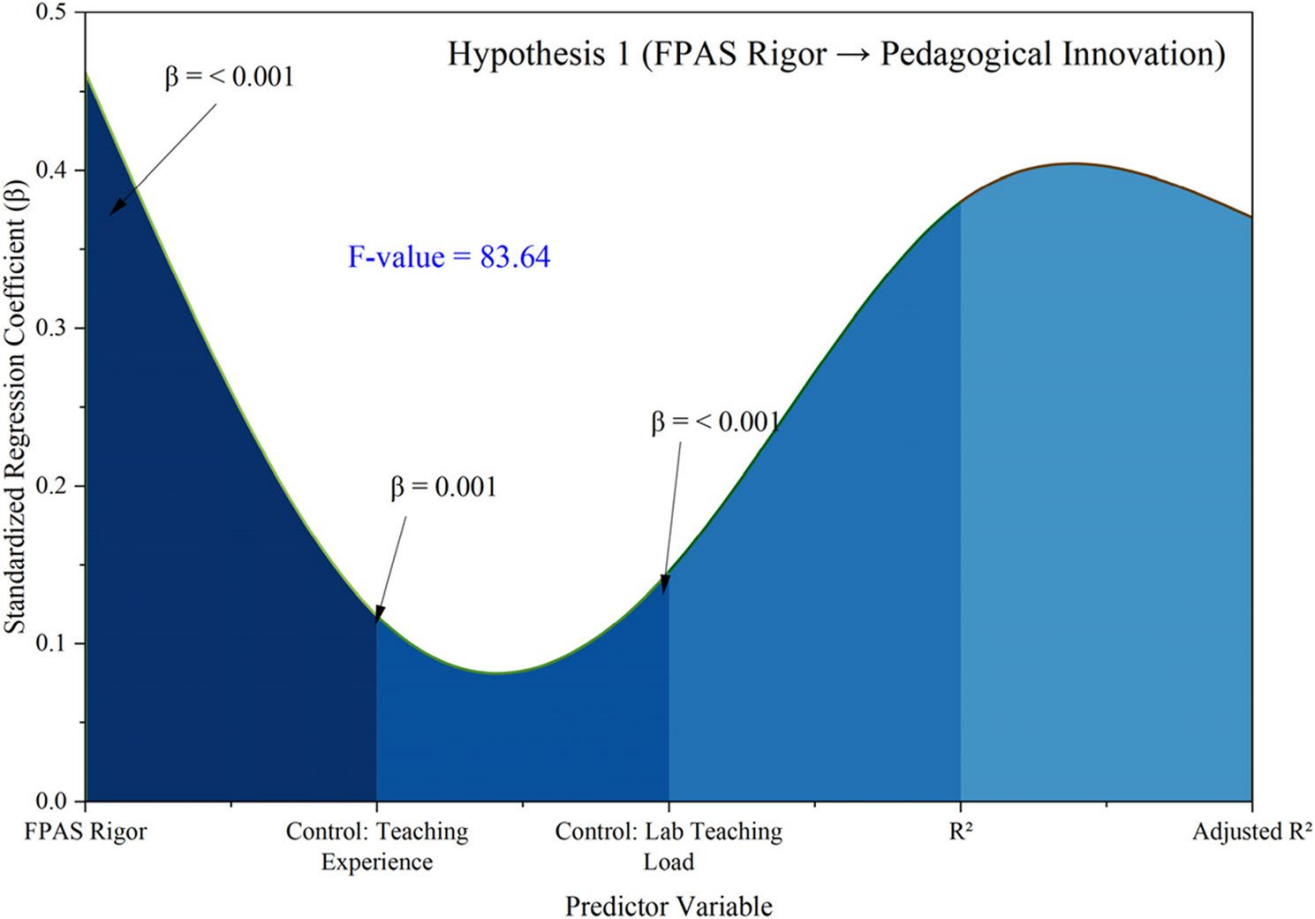
FPAS rigor–pedagogical innovation relationship (H1)

The regression analysis examined the relationship between FPAS rigor and pedagogical innovation while accounting for relevant control variables (Table 3; Fig. 5). Here, the results showed that FPAS Rigor had a strong and statistically significant positive effect on pedagogical innovation (β = 0.462, SE = 0.041, t = 11.27, *p* < 0.001).

**Table 3:** Regression results for hypothesis 1 (FPAS Rigor → pedagogical innovation)

Predictor Variable	β	Std. Error	t-value	*p*-value
FPAS Rigor	0.462	0.041	11.27	< 0.001
Control: Teaching Experience	0.118	0.036	3.28	0.001
Control: Lab Teaching Load	0.146	0.039	3.74	< 0.001
R²	0.38			
Adjusted R²	0.37			
F-value	83.64			< 0.001

The control variables, Teaching Experience (β = 0.118, t = 3.28, *p* = 0.001) and Laboratory Teaching Load (β = 0.146, t = 3.74, *p* < 0.001), also contributed positively and significantly to pedagogical innovation. The model accounted for 38% of the variance in pedagogical innovation (R² = 0.38; Adjusted R² = 0.37), and the overall regression was statistically significant, as indicated by the F-value (F = 83.64, *p* < 0.001).

### Impact of FPAS on laboratory excellence in pharmaceutical sciences

**Fig. 6:**
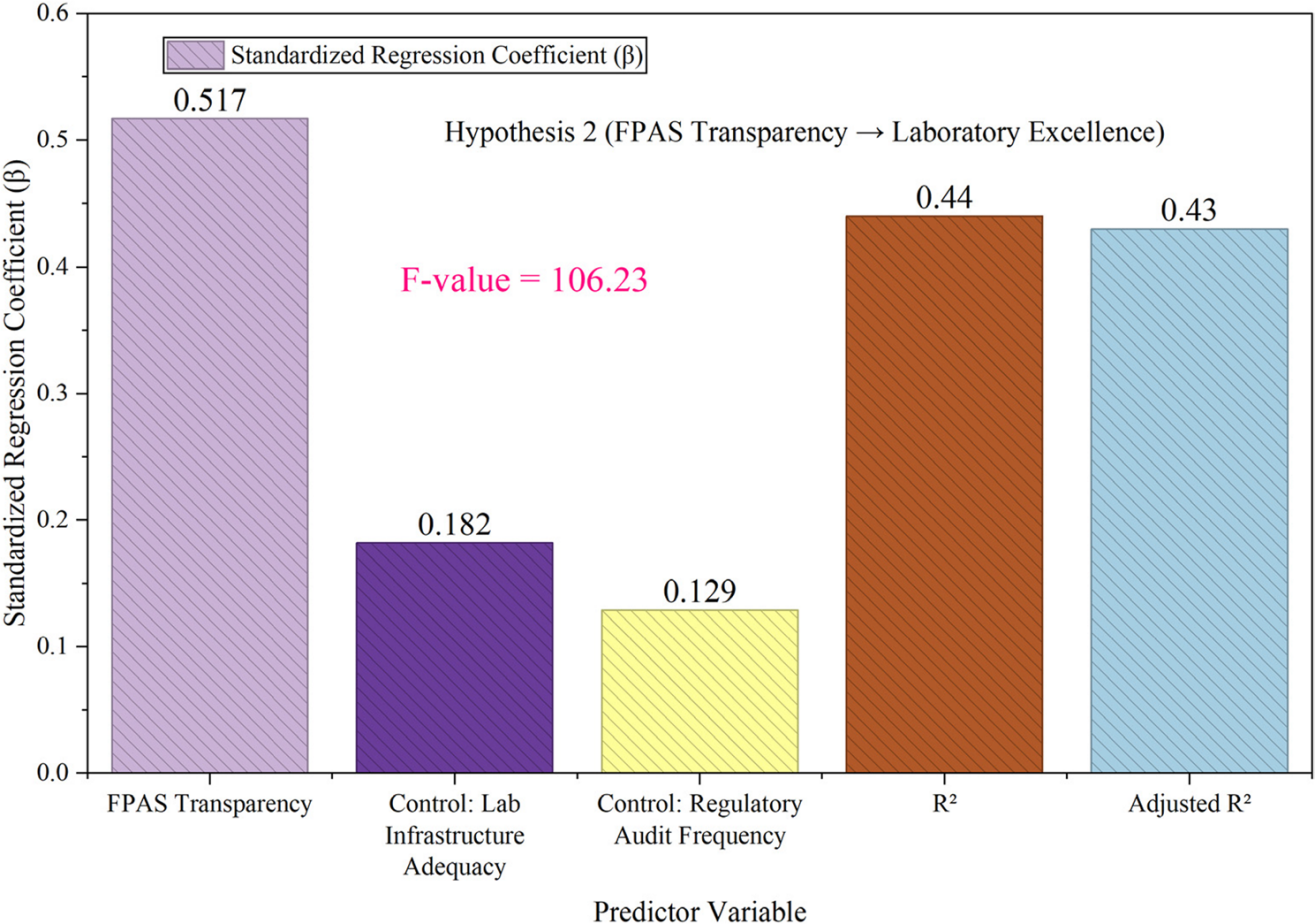
FPAS transparency–laboratory excellence relationship (H2)

Hypothesis 2 was tested to see the relationship between transparency in the process of performance appraisal of the faculty and laboratory excellence in the pharmaceutical education (Table 4; Fig. 6). The empirical results indicated that higher FPAS Transparency was closely related to a higher level of laboratory excellence in terms of a significant standardized coefficient (β = 0.517) and high significance (t = 13.61, *p* < 0.001).

**Table 4:** Hypothesis 3 testing: FPAS transparency and laboratory excellence

Predictor Variable	β	Std. Error	t-value	*p*-value
FPAS Transparency	0.517	0.038	13.61	< 0.001
Control: Lab Infrastructure Adequacy	0.182	0.041	4.44	< 0.001
Control: Regulatory Audit Frequency	0.129	0.034	3.79	< 0.001
R²	0.44			
Adjusted R²	0.43			
F-value	106.23			< 0.001

Also, contextual factors had a significant impact, Laboratory Infrastructure Adequacy (β = 0.182, *p* < 0.001) and Regulatory Audit Frequency (β = 0.129, *p* < 0.001) complemented the results of laboratory quality and compliance. The model had an impressive power to explain (Adjusted R^2^ = 0.43), and the model explained the overall parameter excellence significantly as shown by a large F-statistic (F = 106.23, *p* < 0.001).

### Faculty appraisal and adoption of pharmaceutical education technology

**Fig. 7:**
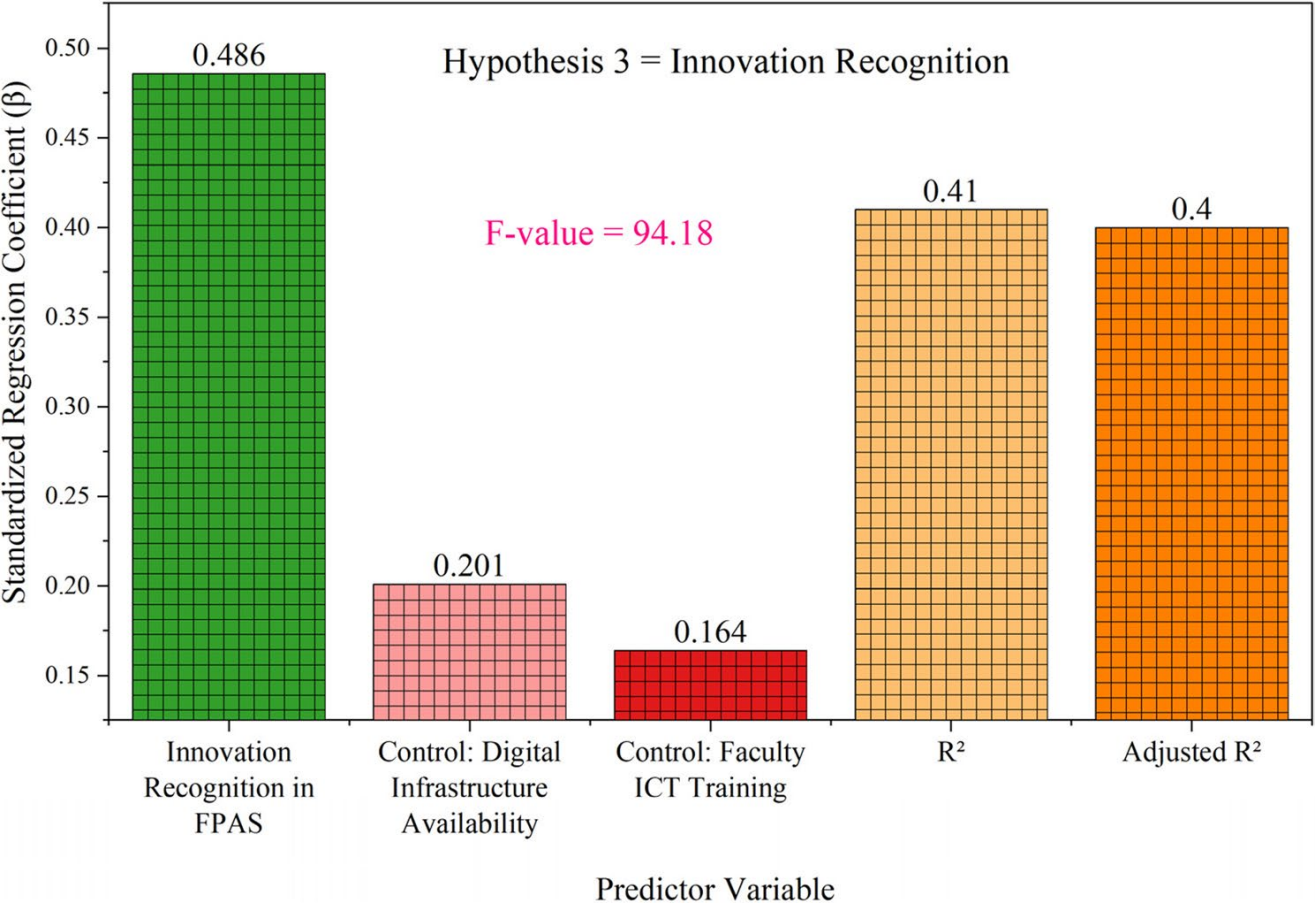
Innovation–recognition linkages in pharmaceutical technology adoption (H3)

Table 5; Fig. 7 showed the regression results of the effect of innovation recognition in the Faculty Performance Appraisal System (FPAS) on the adoption of educational technology in pharmaceutical education. Technology adoption was statistically significantly positively correlated with innovation recognition in FPAS (β = 0.486, *p* < 0.001), which means that the formal recognition of innovative practices positively impacted the use of digital pedagogical tools by the faculty.

**Table 5:** Hypothesis 4 testing: innovation, recognition, and pharmaceutical education technology adoption

Predictor Variable	β	Std. Error	t-value	*p*-value
Innovation Recognition in FPAS	0.486	0.042	11.57	< 0.001
Control: Digital Infrastructure Availability	0.201	0.039	5.15	< 0.001
Control: Faculty ICT Training	0.164	0.037	4.43	< 0.001
R²	0.41			
Adjusted R²	0.40			
F-value	94.18			< 0.001

Digital infrastructure availability (β = 0.201, *p* < 0.001) and faculty ICT training (β = 0.164, *p* < 0.001) control variables also had significant positive relationships with technology adoption, and this indicates the role of institutional readiness and faculty competency development. The regression model accounted 41% percent of the variance in technology adoption (R^2^ = 0.41; Adjusted R^2^ = 0.40), and the overall modelization was justified by a significant F-value (94.18, *p* < 0.001).

### Accreditation pressure, FPAS formalization, and faculty engagement

**Fig. 8:**
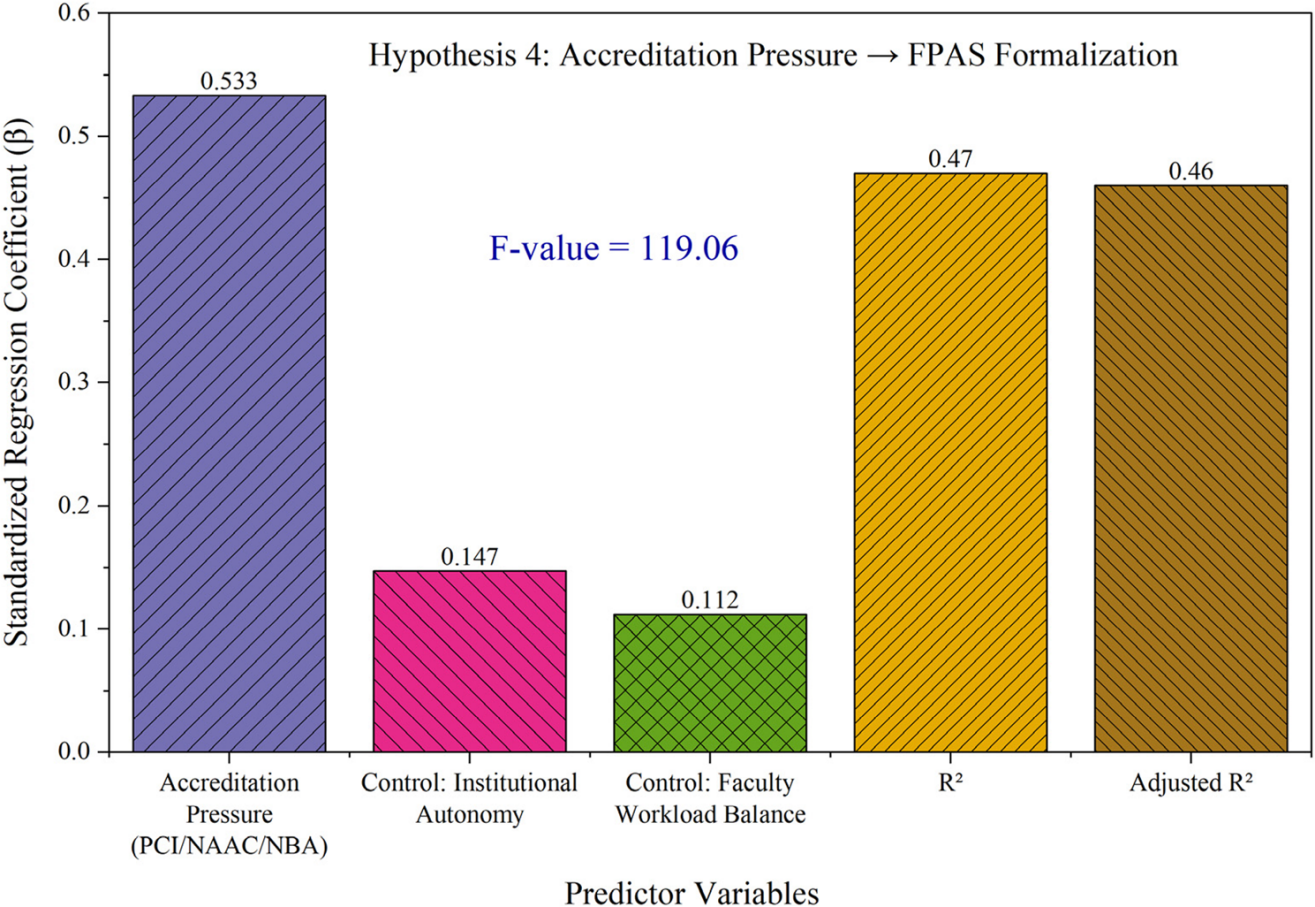
Accreditation–FPAS formalization relationship (H4)

The results of the Hypothesis 5 testing showed that the accreditation pressure exerted by PCI, NAAC and NBA had a strong and statistically significant positive influence on the FPAS formalization ( β = 0.533, t = 14.81, *p* < 0.001) which is shown in (Fig. 8; Table 6). This observation suggested that increased external accreditation demands were linked with more formalized practices of faculty performance appraisal in institutions.

**Table 6:** Hypothesis 4 testing: accreditation pressure and FPAS formalization

Predictor Variable	β	Std. Error	t-value	*p*-value
Accreditation Pressure (PCI/NAAC/NBA)	0.533	0.036	14.81	< 0.001
Control: Institutional Autonomy	0.147	0.035	4.20	< 0.001
Control: Faculty Workload Balance	0.112	0.033	3.39	0.001
R²	0.47			
Adjusted R²	0.46			
F-value	119.06			< 0.001

It also revealed that control variables, institutional autonomy ( 0.147, t = 4.20, *p* = 0.001) and faculty workload balance ( 0.112, t = 3.39, *p* = 0.001) also added significantly and positively to the model. Besides, this model was found to explain 47% of the variance in formalization of FPAS (R 2 = 0.47; adjusted R 2 = 0.46). The overall model fit was statistically significant (F = 119.06, *p* < 0.001), which confirmed the strength of the relationship between accreditation pressure and FPAS formalization.

### FPAS fairness and faculty motivation in pharmaceutical teaching and research

**Fig. 9:**
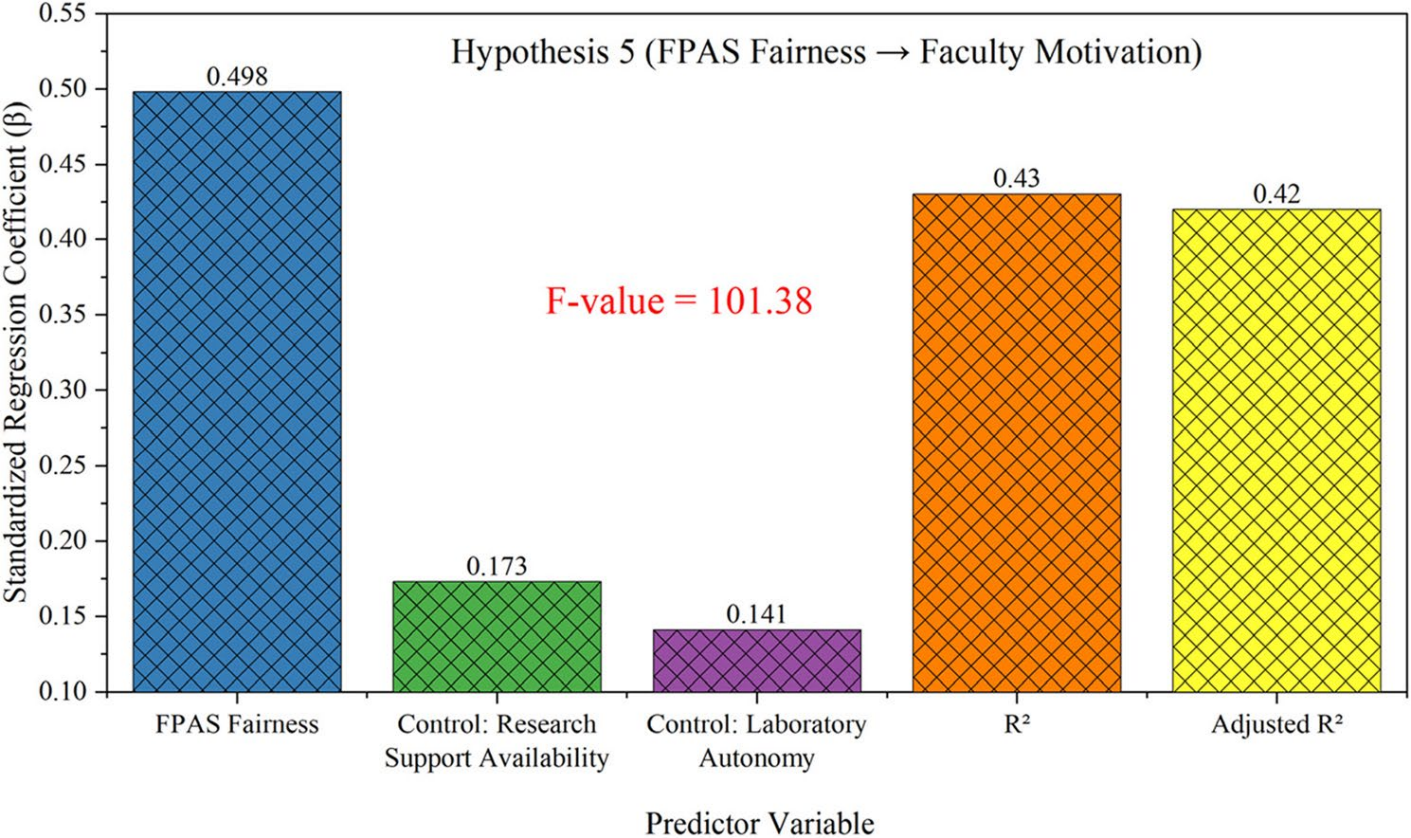
FPAS fairness–faculty motivation relationship (H5)

The regression analysis that was undertaken to investigate the association between FPAS fairness and faculty motivation is presented in Table 7; Fig. 9. There was a significant positive relationship between FPAS fairness and faculty motivation, which means that transparent and fair appraisal processes were relevant to motivational results among the faculty members.

**Table 7:** Hypothesis 6 testing: FPAS fairness and faculty motivation

Predictor Variable	β	Std. Error	t-value	*p*-value
FPAS Fairness	0.498	0.040	12.45	< 0.001
Control: Research Support Availability	0.173	0.038	4.55	< 0.001
Control: Laboratory Autonomy	0.141	0.036	3.92	< 0.001
R²	0.43			
Adjusted R²	0.42			
F-value	101.38			< 0.001

The availability of research and laboratory autonomy also showed significant relationships, and the evidence indicated that the availability of institutional resources and the autonomy of operations helped to maintain the faculty motivation. In general, a significant percentage of variance in faculty motivation was explained by the model, and the regression model proved that the presumed correlation is sufficient.

### Mediating role of laboratory excellence between FPAS and pedagogical innovation

**Fig. 10:**
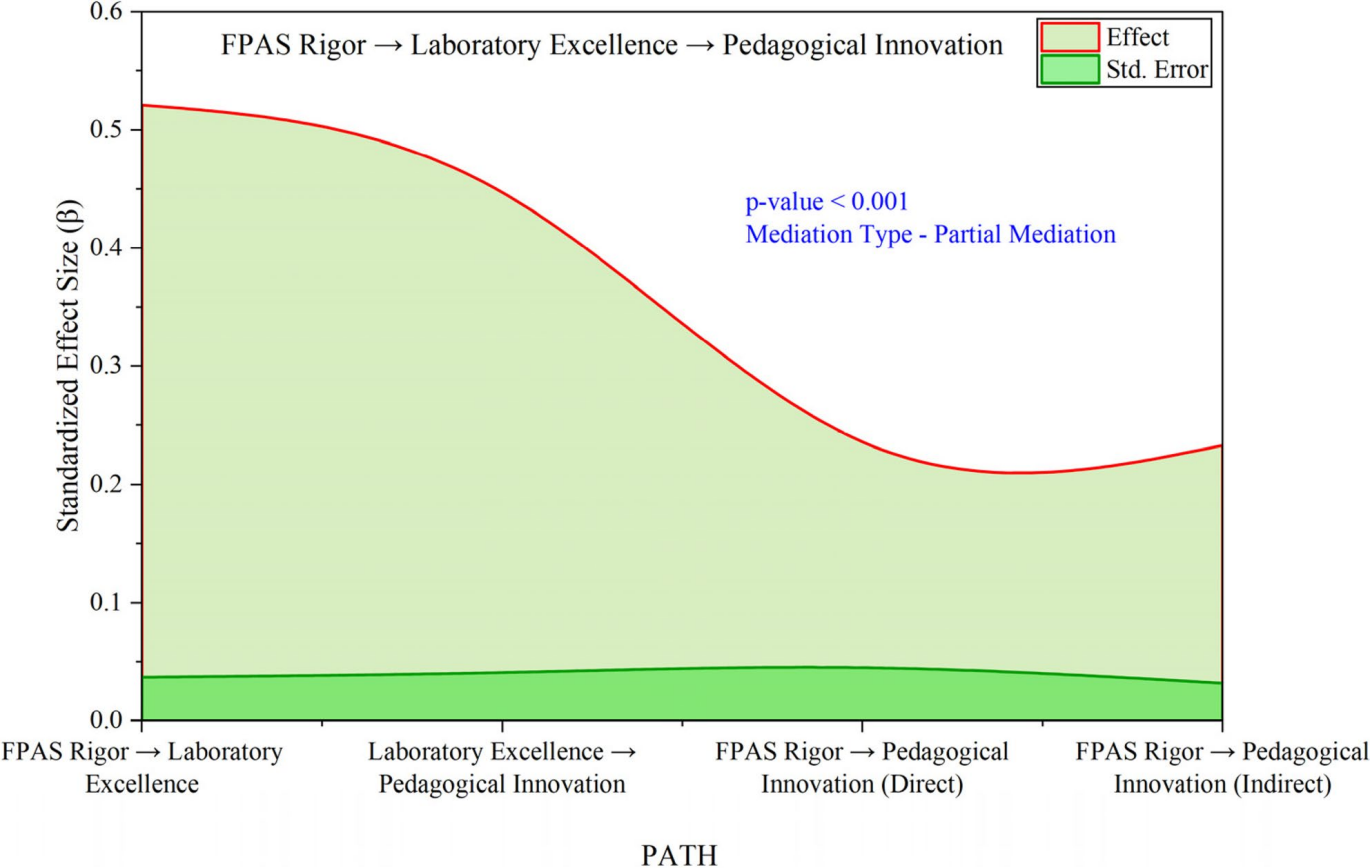
Laboratory excellence in the mediation model

The mediation analysis of the relationship between FPAS rigor and pedagogical innovation that looked at the mediation role of laboratory excellence is reported in Table 8; Fig. 10. There was a positive (significant) impact of FPAS rigor on laboratory excellence (effect = 0.521, *p* < 0.001), and laboratory excellence, in its turn, had a significant (significantly) positive impact on pedagogical innovation (effect = 0.447, *p* < 0.001).

**Table 8:** Mediation analysis: laboratory excellence as a mediator

Path	Effect	Std. Error	t-value	*p*-value
FPAS Rigor → Laboratory Excellence	0.521	0.037	14.08	< 0.001
Laboratory Excellence → Pedagogical Innovation	0.447	0.041	10.90	< 0.001
FPAS Rigor → Pedagogical Innovation (Direct)	0.236	0.045	5.24	< 0.001
FPAS Rigor → Pedagogical Innovation (Indirect)	0.233	0.032	7.28	< 0.001
Mediation Type	Partial Mediation			

﻿The direct relationship between FPAS rigor and pedagogical innovation was statistically significant (effect = 0.236, *p* < 0.001), which meant that FPAS rigor still had an independent effect despite being mediated by the former. The mediating effect via the lab excellence was also important (effect = 0.233, *p* < 0.001), which proved that lab excellence partially mediated the role of FPAS rigor on pedagogical innovation and thus created a partial mediation relationship

### Structural equation modeling of FPAS, laboratory excellence, and pedagogical innovation

**Fig. 11:**
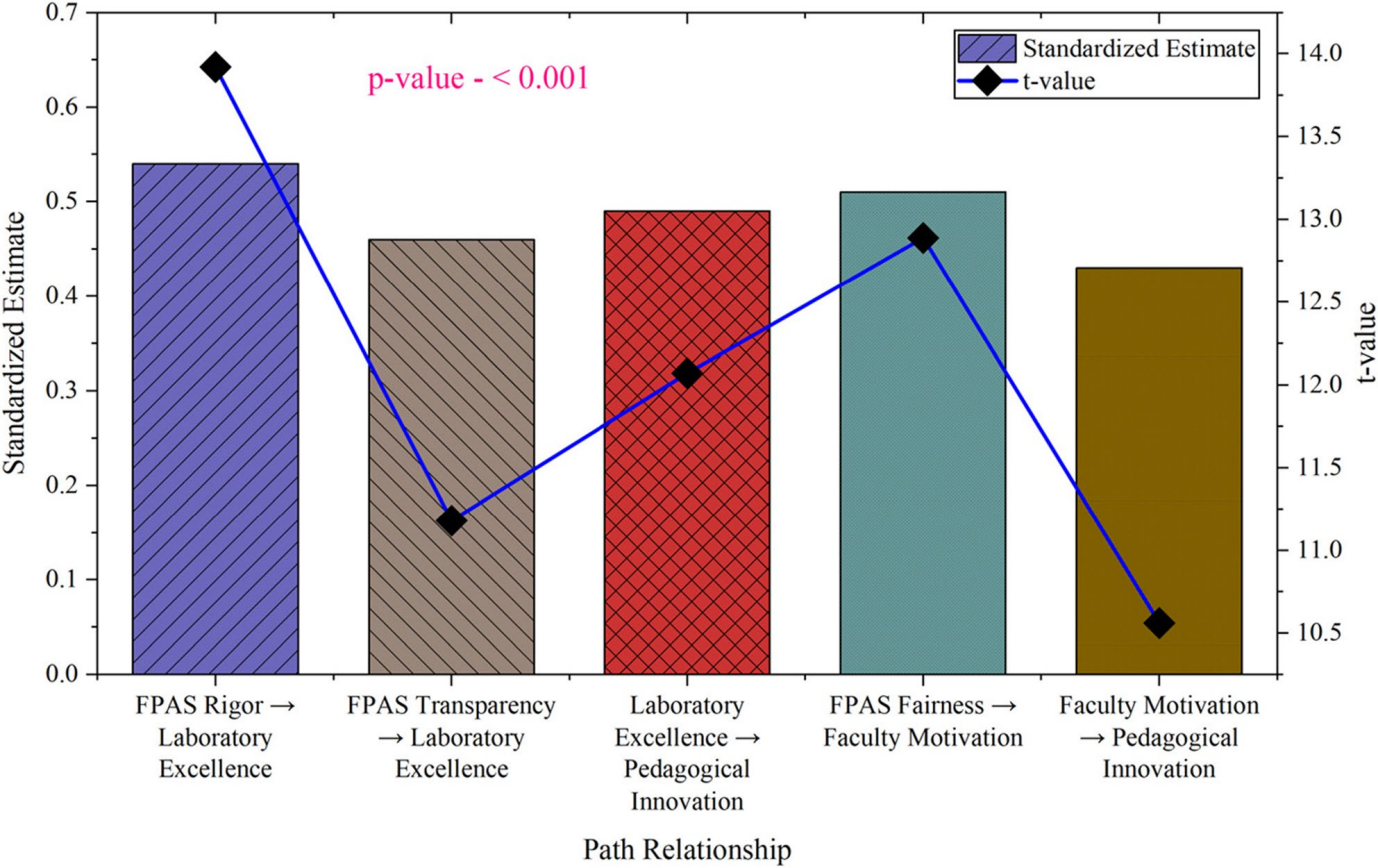
SEM framework for FPAS-driven pedagogical innovation

Table 9; Fig. 11 showed that the structural equation model has statistically significant positive associations between the study variables. The positive impact that rigorous appraisal practices had on laboratory excellence ( 0.54, t = 13.92, *p* < 0.001) suggested that rigorous appraisal practices had a strong positive impact on laboratory excellence. On the same note, transparency of FPAS had a positive impact on laboratory excellence ( 0.46, t = 11.18, *p* < 0.001). In turn, the influence of laboratory excellence had a substantial positive impact on pedagogical innovation (= 0.49, t = 12.07, *p* < 0.001), which implied that a higher standard of laboratories had a significant positive effect on the innovative teaching practices.

**Table 9:** Structural equation model path coefficients and model fit

Path Relationship	Standardized Estimate	t-value	*p*-value
FPAS Rigor → Laboratory Excellence	0.54	13.92	< 0.001
FPAS Transparency → Laboratory Excellence	0.46	11.18	< 0.001
Laboratory Excellence → Pedagogical Innovation	0.49	12.07	< 0.001
FPAS Fairness → Faculty Motivation	0.51	12.89	< 0.001
Faculty Motivation → Pedagogical Innovation	0.43	10.56	< 0.001

These findings also revealed that FPAS fairness had a significant positive impact on faculty motivation (= 0.51, = 12.89, = 0.001), and that faculty motivation also positively influenced pedagogical innovation (= 0.43, = 10.56, = 0.001). The structural model established in Table 9 generally confirmed that rigor, transparency, and fairness in the faculty performance appraisal system were significant predictors of laboratory excellence, faculty motivation and pedagogical innovation.

### Differential effects of FPAS across faculty experience levels in pharmacy institutions

**Fig. 12:**
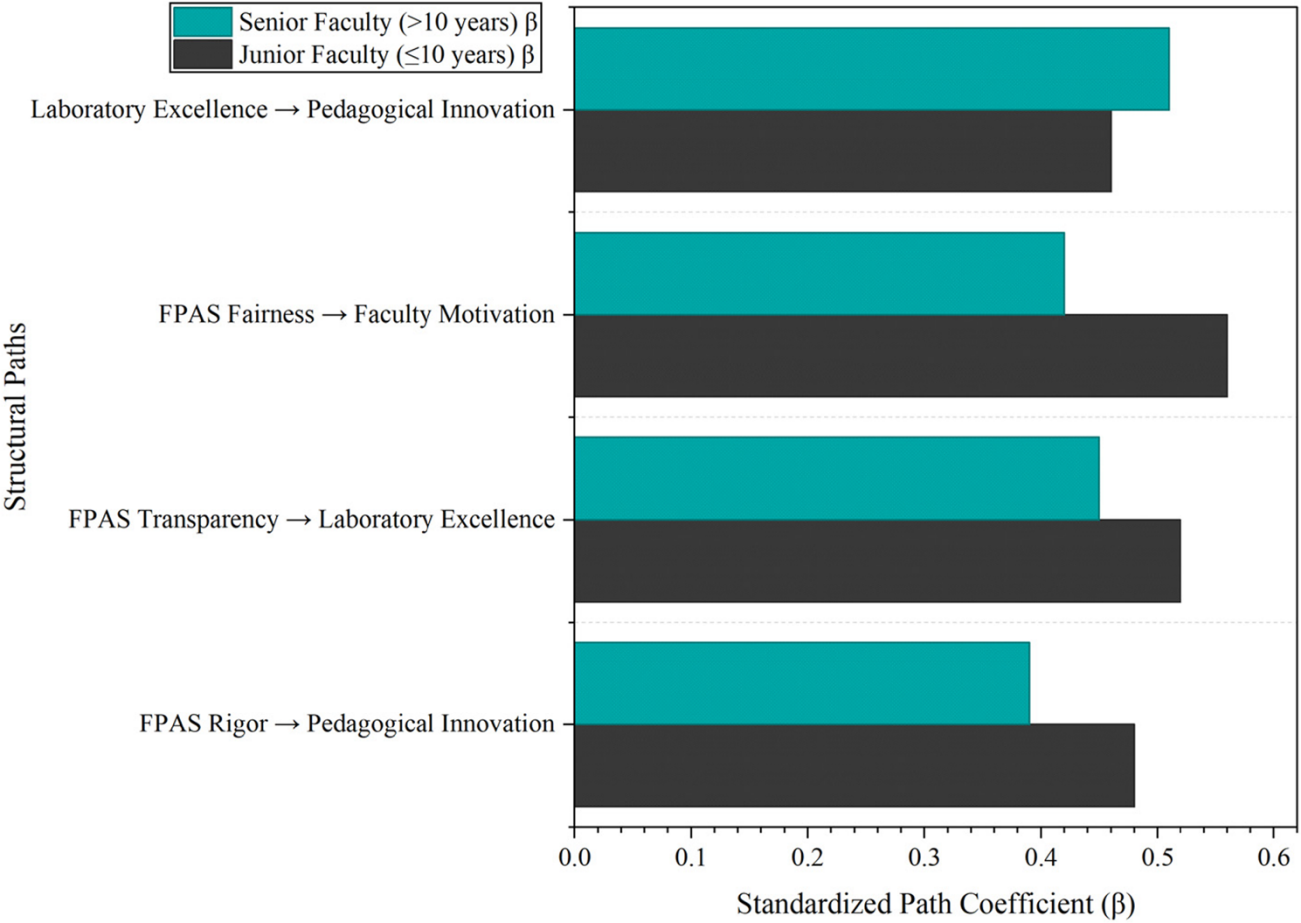
Experience-level moderation of FPAS effects

Table 10; Fig. 12 showed the multigroup analysis of structural path coefficients between the levels of faculty experience. FPAS rigor had a greater influence on pedagogical innovation on junior faculty (β = 0.48) than on senior faculty (β = 0.39), and the observed difference was statistically significant.

**Table 10:** Multi-group analysis by faculty experience level

Path	Junior Faculty (≤ 10 years) β	Senior Faculty (> 10 years) β	Δβ	*p*-value
FPAS Rigor → Pedagogical Innovation	0.48	0.39	0.09	0.021
FPAS Transparency → Laboratory Excellence	0.52	0.45	0.07	0.034
FPAS Fairness → Faculty Motivation	0.56	0.42	0.14	0.008
Laboratory Excellence → Pedagogical Innovation	0.46	0.51	−0.05	0.041

Transparency in FPAS demonstrated a significant relationship with laboratory excellence among junior faculty (β = 0.52) than among senior faculty (β = 0.45), whereas fairness in FPAS had a significant effect on faculty motivation in junior faculty (β = 0.56) than in senior faculty (β = 0.42). Conversely, the direction of laboratory excellence to pedagogical innovation was slightly higher among senior faculty (β = 0.51) compared to junior faculty (β = 0.46), in terms of experience-based variation in the conversion of laboratory quality into pedagogical activities.

### Accreditation-level comparison of FPAS outcomes in pharmaceutical education

**Fig. 13:**
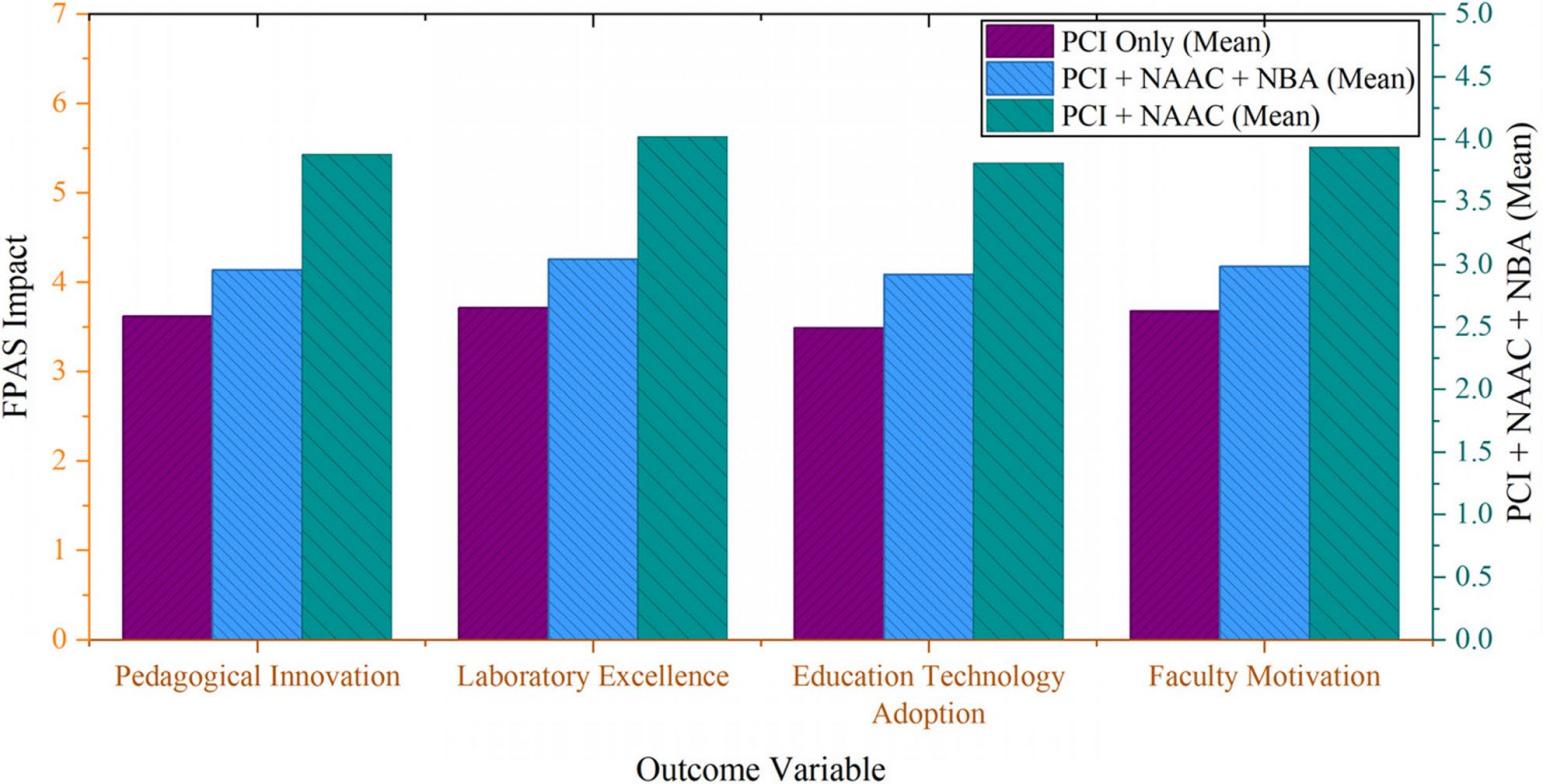
Influence of accreditation level on FPAS outcomes

Table 11; Fig. 13 compared the effect of FPAS outcomes among institutions that were variously exposed to accreditation. The mean scores of pedagogical innovation rose in a progressive manner between PCI-only institutions (3.62) and institutions accredited by PCI, NAAC, and NBA (4.14), which shows that accreditation breadth has a gradual effect. This was also the same trend in laboratory excellence, whose mean values increased between 3.71 and 4.26, indicating improvement in laboratory standards in institutions with multi-accreditation.

**Table 11:** Accreditation-based comparison of FPAS impact

Outcome Variable	PCI Only (Mean)	PCI + NAAC (Mean)	PCI + NAAC + NBA (Mean)	F-value	*p*-value
Pedagogical Innovation	3.62	3.88	4.14	18.96	< 0.001
Laboratory Excellence	3.71	4.02	4.26	22.31	< 0.001
Education Technology Adoption	3.49	3.81	4.09	19.44	< 0.001
Faculty Motivation	3.68	3.94	4.18	17.87	< 0.001

The adoption of education technology also showed incremental gains across accreditation types, with this category going up to 4.09, whereas the faculty motivation showed the same trend and went up to 4.18. The high as well as the statistically significant F-statistics of all outcome variables showed that differences between accreditation groups were not accidental but systematic, which reflects the necessity of cumulative accreditation pressure to influence the creation of FPAS-driven academic and institutional outcomes.

### Summary of hypothesis testing results

The results of hypothesis testing that were performed throughout the study were summarized in Table 12 using various methods of analysis. Each of the hypothesized studies was empirically valid, and the regression analysis proved the direct relationships between the rigor of FPAS and pedagogical innovation, FPAS transparency and laboratory excellence, innovation recognition, education technology adoption, accreditation pressure, and FPAS formalization, and faculty motivation. The mediation theory that investigated the contribution of laboratory excellence in the relationship between FPAS rigor and pedagogical innovation was also confirmed with the help of structural equation modeling and bootstrapping processes. Altogether, the ever-important findings illustrated the internal consistency of the suggested framework as well as the strength of FPAS-related processes in determining pedagogical, technological, and motivational outcomes in pharmaceutical education.

**Table 12:** Summary of hypothesis testing

Hypothesis	Path Tested	Statistical Technique	Result	Significance
H1	FPAS Rigor → Pedagogical Innovation	Regression	Supported	*p* < 0.001
H2	FPAS Transparency → Laboratory Excellence	Regression	Supported	*p* < 0.001
H3	Innovation Recognition → Education Technology Adoption	Regression	Supported	*p* < 0.001
H4	Accreditation Pressure → FPAS Formalization	Regression	Supported	*p* < 0.001
H5	FPAS Fairness → Faculty Motivation	Regression	Supported	*p* < 0.001
H6	Laboratory Excellence → Pedagogical Innovation (Mediation)	SEM / Bootstrapping	Supported	*p* < 0.001

## Discussion

In India, pharmacy education is commonly offered through four-year Bachelor of Pharmacy (B.Pharm) programmes and six-year Doctor of Pharmacy (Pharm.D) programmes, both regulated by the Pharmacy Council of India (PCI). These programmes combine classroom teaching, laboratory-based practical training, and regulatory compliance requirements. Such a structure is relevant to the present study because the laboratory-intensive and accreditation-oriented nature of pharmacy education may shape how faculty performance appraisal systems influence pedagogical innovation, laboratory excellence, and faculty motivation.

The empirical evidence suggests that Faculty Performance Appraisal Systems (FPAS) are significant institutional processes that affect pedagogical innovation, laboratory excellence, and faculty motivation in the field of pharmaceutical education. Regression and structural equation modelling revealed positive relationships between FPAS rigor and transparency and laboratory excellence, which then led to pedagogical innovation. This observation is in line with previous research that has asserted that a well-organized, transparent, and equitable system of faculty evaluation enhances academic engagement, the quality of an institution, and teaching effectiveness in pharmacy education in India [2]. The mediation analysis also revealed that the effect of FPAS rigor upon pedagogical innovation was partially mediated by the laboratory excellence, which showed that appraisal frameworks and real-life teaching settings are tightly interwoven. Other recent studies in the field of pharmacy education have also reported similar observations where institutional support, laboratory infrastructure, and the competency-focused scholarly environments were identified as the important conditions to be improved in order to enhance innovative teaching practices [22].

It was also discovered that FPAS fairness played an important role in faculty motivation, whereas recognition of innovation was found to play a role in adoption of educational technology. Similar results have been found in the recent literature, which identified that faculty members have better responses when appraisal criteria are transparent, role-relevant, and perceived to be fair. Multi-group and accreditation-based analyses further revealed heterogeneity in the effects of FPAS. Junior faculty were more responsive to the rigor, transparency and fairness of appraisal, whereas senior faculty were more capable of translating laboratory excellence into pedagogical innovation. This trend aligns with previous research that has shown a variation in faculty responses based on professional experience and academic role and expectations of the institution [23]. Moreover, the positive changes in the levels of accreditation can support the previous evidence that accreditation requirements have the potential to enhance appraisal practices and facilitate systematic quality improvement in pharmacy education [24].

## Conclusion

The study concluded that the successful implementation of the FPAS models contributed greatly to assessing pedagogical innovation, the quality of labs, the application of technologies, and the motivation of the faculty in pharmaceutical learning institutions within Tamil Nadu. Similar to studies conducted in India, the present study found that structured appraisal systems supported institutional quality and strengthened laboratory-based teaching practices. In comparison with studies reported abroad, the findings also indicate that transparent and performance-oriented appraisal frameworks promoted faculty engagement, innovation adoption, and academic effectiveness in pharmacy education. Faculty Performance Appraisal Systems (FPAS) emerged as a significant institutional mechanism shaping pedagogical innovation, laboratory excellence, technology adoption, and faculty motivation in pharmaceutical education. This paper has found that faculty appraisal in pharmaceutical education is not to be seen simply as an administrative mandate but as an institutional process that has the potential to shape the academic environment in which teaching, lab work, and innovation all develop. The results showed that an appropriately aligned appraisal system that is sensitive to the realities of the profession of pharmacy in its education can lead to increased academic responsiveness, improved institutional coordination and a more significant recognition of faculty contributions beyond the conventional quantitative measures. The research further emphasised the need to contextualise the practices of appraisal within the regulatory and laboratory-intensive nature of pharmaceutical education in Tamil Nadu. Instead of solely using standardized assessment guidelines, organizations can enjoy the advantages of appraisal methods that acknowledge disciplinary demands, faculty functions, and accreditation anticipations. In spite of the fact that the study was confined to pharmacy institutions within Tamil Nadu, it offers a good empirical foundation to future institutional reforms, and to subsequent comparative studies on the role of faculty appraisal in professional higher education.

## Supplementary Information

Supplementary Material 1.

Supplementary Material 2.

Supplementary Material 3.

Supplementary Material 4.

Supplementary Material 5.

## Data Availability

The datasets generated and/or analyzed during the current study are not publicly available due to confidentiality agreements and institutional privacy considerations associated with the participating government-aided and private accredited pharmaceutical educational institutions. However, the data are available from the corresponding author upon reasonable request for academic and research purposes.

## Abbreviations

FPAS: Faculty Performance Appraisal System
NEP: National Education Policy
PCI: Pharmacy Council of India
APC: Article Processing Charges
CBE: Competency-Based Education
TPACK: Technological Pedagogical Content Knowledge
LMS: Learning Management System
ICT: Information and Communication Technology
CPD: Continuing Professional Development
CE: Continuing Education
PBL: Problem-Based Learning
TEL: Technology-Enhanced Learning
HEd: Higher Education
IJPER: Indian Journal of Pharmaceutical Education and Research
BMC: BioMed Central
MSc: Master of Science
PhD: Doctor of Philosophy
AI: Artificial Intelligence
OBE: Outcome-Based Education
QA: Quality Assurance
