# Supplementary material for: Evaluating the impact of faculty performance appraisal systems on curriculum development and laboratory innovation in pharmaceutical education
Source: BMC Med Educ. 2026 Jun 26;26:1200. doi: 10.1186/s12909-026-09744-0 (PMC13393486; doi:10.1186/s12909-026-09744-0)
Supplement: Supplementary file 1 — Supplementary Material 1. [file 12909_2026_9744_MOESM1_ESM.docx]

**Research Highlights**

- Identifies critical gaps in existing Faculty Performance Appraisal Systems (FPAS) within pharmaceutical education in Tamil Nadu.
- Demonstrates how conventional appraisal frameworks undervalue laboratory innovation and educational technology integration.
- Reveals the impact of subjective and quantitative-heavy evaluations on faculty motivation and pedagogical stagnation.
- Proposes a redesigned, transparent, and multidimensional appraisal model tailored to modern pharmacy curricula.
- Emphasizes incentivization of **Pharmaceutical Education Technology** and **laboratory excellence** as drivers of teaching innovation.
- Provides policy-relevant insights to support faculty development and curriculum advancement in pharmaceutical education.
