# Supplementary material for: Evaluating the impact of faculty performance appraisal systems on curriculum development and laboratory innovation in pharmaceutical education
Source: BMC Med Educ. 2026 Jun 26;26:1200. doi: 10.1186/s12909-026-09744-0 (PMC13393486; doi:10.1186/s12909-026-09744-0)
Supplement: Supplementary file 2 — Supplementary Material 2. [file 12909_2026_9744_MOESM2_ESM.pdf]

Evaluating Faculty Performance Systems for Educational Quality Enhancement in Pharmacy Institutions

Questionnaire Title:

Faculty Performance Appraisal, Curriculum Innovation, and Laboratory Development in Pharmacy Education Institutions of Tamil Nadu

Purpose:

This questionnaire aims to evaluate faculty perceptions of existing Faculty Performance Appraisal Systems (FPAS) and their alignment with curriculum innovation, technology-enhanced teaching, and laboratory modernization in pharmacy institutions.

Section A: Demographic Information

1. Gender: ☐ Male ☐ Female ☐ Prefer not to say
2. Age Group: ☐ Below 30 ☐ 31–40 ☐ 41–50 ☐ Above 50
3. Highest Qualification: ☐ M.Pharm ☐ Ph.D. ☐ Post-Doctoral ☐ Other: ______
4. Designation: ☐ Lecturer ☐ Assistant Professor ☐ Associate Professor ☐ Professor
5. Years of Teaching Experience: ☐ <5 ☐ 5–10 ☐ 11–15 ☐ >15
6. Type of Institution: ☐ Autonomous ☐ Affiliated ☐ Deemed University ☐ Private
7. Area of Specialization: ______________________

Faculty Performance Appraisal Questionnaire in Pharmacy Education

Response Scale:

1 – Strongly Disagree

| 2 – Disagree

| 3 – Neutral

| 4 – Agree

| 5 – Strongly Agree

Section B: Existing Faculty Performance Appraisal System (FPAS)

No.	Statement	1	2	3	4	5
1	The current FPAS in my institution clearly defines evaluation criteria.	☐	☐	☐	☐	☐
2	FPAS primarily emphasizes quantitative outputs such as publications and pass percentage.	☐	☐	☐	☐	☐
3	Subjective judgments significantly influence appraisal outcomes.	☐	☐	☐	☐	☐
4	FPAS outcomes contribute to professional growth and motivation.	☐	☐	☐	☐	☐
5	The appraisal process is transparent and fair.	☐	☐	☐	☐	☐

Section C: Curriculum Innovation and Teaching Practices

No.	Statement	1	2	3	4	5
6	Curriculum redesign activities are formally recognized in FPAS.					
7	Innovative teaching approaches such as ICT tools, simulation, and blended learning are valued.					
8	Outcome-Based Education implementation is encouraged by the institution.					
9	Institutional support exists for pedagogical experimentation and new course models.					
10	Teaching innovation influences promotion, incentives, or recognition.					

Section D: Laboratory Development and Accreditation Integration

No.	Statement	1	2	3	4	5
11	Laboratory modernization efforts are considered within FPAS evaluation.					
12	Faculty contributions to laboratory setup, validation, or upgrading are recognized.					
13	Digital or virtual laboratory tools are encouraged in teaching practice.					
14	Accreditation requirements significantly influence laboratory improvement.					
15	Integration of laboratory-based research into teaching is valued by the institution.					

Section E: Faculty Motivation and Institutional Impact

No.	Statement	1	2	3	4	5
16	Misalignment in FPAS reduces faculty motivation for innovation.					
17	Recognition of innovation would improve overall teaching quality.					
18	Multidimensional appraisal systems are necessary in pharmacy education.					
19	FPAS reform would enhance industry relevance of the curriculum.					
20	Transparent appraisal practices strengthen institutional academic culture.					

Section F: Open-Ended Responses

21. What major limitations do you observe in the current FPAS?

22. What appraisal indicators should be added to support curriculum and laboratory innovation?

23. Additional suggestions for improving pharmacy education quality:
