# Supplementary material for: Evaluating the impact of faculty performance appraisal systems on curriculum development and laboratory innovation in pharmaceutical education
Source: BMC Med Educ. 2026 Jun 26;26:1200. doi: 10.1186/s12909-026-09744-0 (PMC13393486; doi:10.1186/s12909-026-09744-0)
Supplement: Supplementary file 3 — Supplementary Material 3. [file 12909_2026_9744_MOESM3_ESM.pdf]

SETHU INSTITUTE OF TECHNOLOGY

AN AUTONOMOUS INSTITUTION | ACCREDITED WITH 'A++' GRADE BY NAAC

(Approved by AICTE, New Delhi & Affiliated to Anna University, Chennai.)
Recognized by UGC Under Section 2(f) & 12(B) of UGC Act, 1956
Approved Research Centre for B.E. Mech., CSE, ECE, EEE, CSE(AI& ML), B.Tech. IT, CSBS, Physics & Chemistry by Anna University, Chennai.
B.E. Mech., CSE, ECE, EEE, Civil, BME, B.Tech. IT, M.E. CAD/CAM, COS,CSE, PED & Stru.Engg. Programmes are Accredited by NBA, New Delhi under Tier-1(Washington Accord)

Estd : 1995

Pulloor - 626 115, Kariapatti Taluk, Virudhunagar District, Tamil Nadu, India.
Phone : 04566 - 229706   Mobile : 99433 67007
Website : www.sethu.ac.in   Email : principal@sethu.ac.in

TO WHOMSOEVER IT MAY CONCERN

This is to certify that **Ms. Seeni Mohamed Jaleel Nilofer Fathima,** Assistant Professor, Department of Science & Humanities, **Sethu Institute of Technology**, Virudhunagar, Tamil Nadu, **India – 626115.** has conducted a research study involving voluntary participation through a structured questionnaire survey.

This study was reviewed and approved by the Institutional Ethics Committee (IEC) of Sethu Institute of Technology, India (Approval No.: SIT/IEC/2025/1302). All procedures performed in this study were in accordance with the ethical standards of the institutional research committee and with the 1964 Helsinki Declaration and its later amendments. Written informed consent was obtained from all participants prior to their inclusion in the study.

The study does not involve any clinical intervention, biological sample collection, or sensitive personal identification data. Participation was entirely voluntary, and informed consent was obtained from all respondents prior to data collection. The research has been carried out in accordance with accepted ethical standards and the principles of the Declaration of Helsinki.

All participants were informed about the purpose of the research, and their participation was entirely voluntary. Written informed consent was obtained from all respondents prior to their inclusion in the study and before data collection.

This certificate is issued for academic and publication purposes upon request.

SETHU INSTITUTE OF TECHNOLOGY ★ PULLOOR KARIAPATTI 626 115

Head of the Institution / Authorized Signatory
Sethu Institute of Technology
Virudhunagar, Tamil Nadu – 626115, India

PRINCIPAL
SETHU INSTITUTE OF TECHNOLOGY
PULLOOR, KARIAPATTI - 626 115
VIRUDHUNAGAR (DT)

Date: 13 – 02 – 2026
Place: Virudhunagar

Scanned with OKEN Scanner
